# Phenolic Content, Main Flavonoids, and Antioxidant Capacity of Instant Sweet Tea (*Lithocarpus litseifolius* [Hance] Chun) Prepared with Different Raw Materials and Drying Methods

**DOI:** 10.3390/foods10081930

**Published:** 2021-08-19

**Authors:** Hong-Yan Liu, Yi Liu, Ying-Hui Mai, Huan Guo, Xiao-Qin He, Yu Xia, Hang Li, Qi-Guo Zhuang, Ren-You Gan

**Affiliations:** 1 Research Center for Plants and Human Health, Institute of Urban Agriculture, Chinese Academy of Agricultural Sciences, Chengdu 610213, China; liuhongyan01@caas.cn (H.-Y.L.); liuyi03@caas.cn (Y.L.); ghscny@163.com (H.G.); 18883394393@163.com (X.-Q.H.); xiayu01@caas.cn (Y.X.); tiantsai@sina.com (H.L.); 2 Chengdu National Agricultural Science & Technology Center, Chengdu 610213, China; 3 School of Chemical Science, University of Auckland, Auckland 1142, New Zealand; myin315@aucklanduni.ac.nz; 4 China-New Zealand Belt and Road Joint Laboratory on Kiwifruit, Kiwifruit Breeding and Utilization Key Laboratory of Sichuan Province, Sichuan Provincial Academy of Natural Resource Sciences, Tianfu New Area, Chengdu 610213, China; qgzhuang@126.com; 5 Key Laboratory of Coarse Cereal Processing (Ministry of Agriculture and Rural Affairs), Sichuan Engineering & Technology Research Center of Coarse Cereal Industralization, College of Food and Biological Engineering, Chengdu University, Chengdu 610106, China

**Keywords:** *Lithocarpus litseifolius*, instant tea, phenolics, antioxidant, response surface methodology

## Abstract

This study aims to investigate the effects of raw materials and drying methods on the phytochemical and antioxidant capacities of instant sweet tea powder. Four raw materials of sweet tea leave powders (STUT) were extracted and dried with two methods (freeze-drying and spray-drying). The antioxidant capacity, total phenolic content (TPC), total flavonoid content (TFC), and phlorizin and trilobatin contents of obtained instant sweet tea powders were compared. In addition, the single-factor experiments coupled with response surface methodology were used to study the influences of solvent-to-sample ratio, extraction temperature, extraction time, and their interactions on instant sweet tea yield. Results showed that the optimal conditions for extraction were the solvent-to-sample ratio of 19:1 mL/g, extraction temperature of 88 °C, and extraction time of 30 min. The TPC, TFC, antioxidant capacities, and phloridzin and trilobatin contents of instant sweet teas were higher than those of STUT, and the TPC and TFC of freeze-dried instant sweet teas were higher than those of spray-dried instant sweet teas. Significant correlations were found among TPC, TFC, and antioxidant capacities (*p* < 0.01). The freeze-dried instant sweet tea produced by young leaves (prepared by oven-drying) showed the highest TPC, TFC, and antioxidant capacities, compared with other raw materials and drying methods.

## 1. Introduction

*Lithocarpus litseifolius* [Hance] Chun, also popularly called “sweet tea”, is a herbal plant with an evident sweet taste that has been accepted as a daily beverage in the south of China for more than 1000 years, and was approved as a new food material in China in 2017 [1,2]. The major bioactive components in sweet tea include flavonoids and polysaccharides; dihydrochalcones are the main flavonoids [3,4]. Until now, many health benefits have been reported of sweet tea, such as antioxidant, antihypertensive, antihyperlipidemic, antihyperglycemic, anti-inflammatory, antimicrobial, and anticancer effects [1,5]. Instant tea powder is a kind of soluble solid tea produced by extracting tea leaves and drying the tea infusion. Instant teas produced from green, black, and dark teas have been studied by many researchers, while seldom studies have reported the instant sweet teas. Instant teas are commonly extracted by hot water and concentrated by vacuum/reverse osmosis, and further dried by spray-, freeze-, or vacuum-drying methods [6]. Previous studies demonstrated that sweet tea and its dihydrochalcones showed antioxidant activity [7]. Whether these processes during instant sweet tea production can affect the phytochemicals and antioxidant capacity of sweet tea products is still unclear.

Most instant teas are usually produced by ‘broken mixed fannings’ (BMF) because it is cheap, and different raw materials have been developed for instant tea production in recent years [8]. The effects of raw materials on the antioxidant capacity and phytochemicals of the final instant teas are not uniform. Total phenolics and antioxidant activities of instant teas were not significantly changed by the quality of black teas [9]. However, other researchers found that instant black teas prepared by fermented dhools showed higher polyphenolic and theaflavin contents, as well as antioxidant activities than those prepared by black tea and BMF [8]. In addition, the extraction/brew conditions (e.g., extraction temperature and time, brewing time, and water volume) were found to significantly influence the total soluble solid, phenolic content, and antioxidant activity of instant teas [10,11,12,13,14].

Two main drying methods, including spray-drying and freeze-drying, are commonly used for instant tea production. The spray-drying method is a conventional and economic process for commercial instant tea production, while the instant tea produced by freeze-drying exhibits more aromatic compounds in comparison with that produced by spray-drying [15]. The former dried the liquid and semi-liquid by inlet hot air while the latter was operated at low temperatures. With different drying methods or temperature, the final products can present different bioactive and aromatic compounds, as well as different sensory qualities [9,15,16].

In view of the above, different processing methods can lead to different qualities of instant teas, therefore, it is essential to investigate the effects of extraction and drying methods on their main properties. In this study, single-factor tests and response surface methodology (RSM) were conducted to obtain the maximum yield of instant sweet tea powder by optimizing the extraction conditions. Furthermore, the antioxidant activity, total phenolic content (TPC), total flavonoid content (TFC), and phloridzin and trilobatin contents of *Lithocarpus litseifolius* [Hance] Chun and its instant tea powder samples were assessed under the optimal conditions. Finally, the relationships among antioxidant capacity and phytochemical contents were investigated. It is expected that the outcome of this study may provide references for improving the quality of instant sweet tea processing.

## 2. Materials and Methods

### 2.1. Samples and Reagents

In the summer of 2020, young (10–13 days) and old leaves (17–20 days) of *Lithocarpus litseifolius* [Hance] Chun were collected from Ya’an, Sichuan Province, China. The leaves were, respectively, dried by an oven (DHG-9246A, Shanghai Jinghong Experimental Equipment Co., Ltd., China) at 40 °C for 72 h and a microwave (XH-6KW, Xinhong, China) at 450 °C for 4 min to obtain four kinds of raw materials, including microwave-dried young leaves (MY), microwave-dried old leaves (MO), oven-dried young leaves (OY), and oven-dried old leaves (OO). The leaves were, respectively, ground with a Cyclotec 1093 sample mill (Foss Tecator, Denmark) and then were sieved by a mesh with 0.075 mm. Four untreated sweet tea (STUT) samples from *Lithocarpus litseifolius* [Hance] Chun leaves (MY, MO, OY, and OO) were obtained.

The chemicals, including Folin–Ciocalteu’s phenol reagent, 6-hydroxy-2,5,7,8-tetramethylchromane-2-carboxylic acid (Trolox), 2,2′-azinobis (3-ethylbenothiazoline-6-sulfonic acid) (ABTS), 2,4,6-tri(2-pyridyl)-S-triazine (TPTZ), gallic acid, and catechin were purchased from Sigma-Aldrich (St. Louis, MO, USA). The standards of phloridzin and trilobatin were purchased from Madsen Technology Co., Ltd. (Chengdu, China). The formic acid, ethanol, and methanol were obtained from Shanghai Macklin Biochemical Co., Ltd. (Shanghai, China). The 2,2-diphenyl-1-picrylhydrazyl (DPPH), sodium acetate, ferric chloride, and Na_2_CO_3_ (>99%) were purchased from Beijing Solarbio Technology Co., Ltd. (Beijing, China).

### 2.2. Optimization of Instant Sweet Tea Powder Extraction

The young leaf powders dried by microwave (MY) were used to conduct an extraction optimization experiment. The obtained suspension was centrifuged at 3000*× g* for 20 min, and a rotary evaporator (RV-8, IKA Instrument Co., Ltd., Staufen, Germany) was used to concentrate the supernatant of STUT. The concentrates were further freeze-dried by a freeze-dryer (SJIA-5S, Ningbo Shuangjia Instrument Co., Ltd., China) from −40 to 25 °C for 72 h, and the yield (g/100 g) of instant sweet tea powder was calculated.

#### 2.2.1. Single-Factor Tests

The effects of three factors on extraction efficiency were evaluated, including solvent-to-sample ratio (10:1, 15:1, 20:1, 25:1, and 30:1 mL/g), extraction time (20, 30, 40, 50, and 60 min), and extraction temperature (55, 65, 75, 85, and 95 °C). The effect on the yield (g/100 g) of instant sweet tea powder was investigated by changing one factor and fixing the other two parameters.

#### 2.2.2. Response Surface Methodology (RSM)

A three-level-three-variables Box–Behnken Design (BBD) was conducted in order to optimize the extraction condition of instant sweet tea powder. The three major variables included solvent-to-sample ratio (15:1, 20:1, and 25:1), extraction temperature (75, 85, and 95 °C) and extraction time (30, 40, and 50 min), and each variable was encoded as −1, 0, and 1 denoting the low, mid, and high levels. The BBD matrix contained 17 experiments with five replicates of the center points.

### 2.3. Preparation of Instant Sweet Tea Powder with Different Raw Materials and Drying Methods

Four raw materials (STUT) were extracted with hot water at the optimal extraction condition determined with RSM, and the obtained suspension was centrifuged as described in Section 2.2. The concentrate was, respectively, freeze-dried and spray-dried by a spray dryer (JT-6000Y, Hangzhou Jutong Electronic Co., Ltd., Hangzhou, China). The condition for freeze-drying was from −40 to 25 °C for 72 h, while the inlet air temperature of spray-drying was 70 °C, and the feed rate was 400 mL/h. Finally, instant sweet tea powder samples obtained by freeze-drying (STFD) and spray-drying (STSD) were prepared from each raw material. The STUT, STFD, and STSD powder samples were stored at −20 °C until the analysis.

### 2.4. Determination of TPC and TFC

Four raw materials of sweet tea leaf powder samples and eight instant sweet tea powder samples produced as described in Section 2.1 and Section 2.3 were analyzed. The sample (1.000 g) was immersed in 15 mL of 60% (*v/v*) ethanol aqueous solution and extracted in a water bath shaker at 25 °C for 24 h. After centrifugation (3000× *g*, 15 min), the supernatant was collected and diluted by 60% of ethanol before the measurement. All the contents were finally corrected with the dilution fold.

The TPC was detected by the Folin–Ciocalteu method [17], as reported previously, at room temperature. The 400 μL diluted sample solution was mixed with 2 mL Folin–Ciocalteu reagent for 4 min, and then 1.6 mL of saturated Na_2_CO_3_ solution was added. The mixture was kept in dark conditions for 2 h, and the absorbance of the mixture was determined at 760 nm by a UV–visible spectrophotometer (UV1800, Jinghua Instrument Co., Ltd., Shanghai, China). The gallic acid standard curve was drawn, and the TPC content was expressed by mg gallic acid equivalent (GAE)/g dry weight (DW). Each sample was determined in triplicate.

The TFC was determined according to a previous report [18,19] with slight modification. The diluted sample solution (0.5 mL), distilled water (3.5 mL), and 5% NaNO_2_ solution (150 μL) were mixed and shaken. Then, 150 μL of 10% AlCl_3_ were added and reacted for 6 min before adding 1 mL of 1.0 M NaOH. The mixture was left at ambient temperature for 15 min, and the absorbance was detected at 510 nm. The catechin standard curve was drawn, and TFC was expressed as mg catechin equivalent (CE)/g DW of samples. Each sample was carried out in triplicate.

### 2.5. Determination of Antioxidant Capacity

The sample extraction and dilution methods were the same as the methods for determining the TPC and TFC; all the values were finally corrected with the dilution fold.

#### 2.5.1. Determination of DPPH Radical Scavenging Activity

The DPPH radical scavenging activity was analyzed based on previous literature [20,21]. The DPPH working solution was prepared by diluting the DPPH stock solution (100 μM) with 80% methanol to obtain an absorbance of 0.70 ± 0.05 at 515 nm. For the sample determination, 100 μL of the diluted sample solution were added to 3.9 mL of the DPPH working solution for 120 min in dark, and the absorbance was further determined at 515 nm. Obtained results were expressed as µmol of Trolox/g dry weight (DW).

#### 2.5.2. Determination of Ferric-Reducing Antioxidant Power (FRAP Assay)

The FRAP assay was determined according to a previous study [22]. The FRAP working solution consisted of 300 mmol/L sodium acetate buffer (pH 3.5), 10 mmol/L TPTZ solution, and 20 mmol/L ferric chloride solution (10:1:1, *v/v/v*). On determination, 100 μL of the properly diluted supernatant was added to 3 mL of the FRAP working solution for 4 min at room temperature, and then the absorbance was detected at 593 nm. The ferrous sulfate standard curve was drawn, and the FRAP value was expressed as µmol Fe(II)/g dry weight (DW).

#### 2.5.3. Determination of ABTS Cation Radical Scavenging Activity

The ABTS cation radical scavenging activity was measured based on previous literature [23]. The 7 mmol/L ABTS solution and 2.45 mmol/L potassium persulfate solution were mixed at a volume ratio of 1:1 to obtain the ABTS stock solution. Then, the stock solution was used after 16–24 h of light-avoiding incubation. The ABTS working solution was prepared by diluting ABTS stock solution to ensure its absorbance of 0.70 ± 0.05 at 734 nm. On determination, 3.9 mL of the ABTS working solution was added into 100 μL of the properly diluted supernatant and thoroughly mixed, and the reaction was conducted to protect from light and kept for 6 min at room temperature. Then, the absorbance of the mixture was determined at 734 nm. The Trolox solution standard curve was drawn, and the final ABTS value was expressed by μmol Trolox/g dry weight (DW).

### 2.6. HPLC Analysis of Phlorizin and Trilobatin

High-performance liquid chromatography (HPLC) analysis with Agilent 1260 II HPLC system coupled with a DAD detector was employed to identify and quantify dihydrochalcones in instant sweet teas, and the Agilent Zorbax SB-C18 column (4.6 × 150 mm, 5 µm) was used at 30 °C. Water containing 0.1% formic acid (solvent A) and acetonitrile (solvent B) constituted the mobile phase. The gradient program was as follows: 5~60% B, 0–10 min; 60~85% B, 10–15 min; 85~100% B, 15–20 min; 100% B, 20–25 min; 100~5% B, 25–30 min; and 5% B, 30–35 min. Tea powder samples were dissolved with 70% ethanol and filtered through a 0.22 μm filter before injection. The injection volume was 10 μL and the flow rate was 1.0 mL/min. Phloridzin and trilobatin peaks were monitored at 280 nm and identified by comparing their retention time with respective standard. Data were expressed as mg/g of dry weight (DW).

### 2.7. Statistical Analysis

The response values of the RSM model were analyzed by Design-Expert 8.0.6. (Stat-Ease Inc., Minneapolis, MN, USA). SPSS 18.0 statistics software (SPSS Inc., Chicago, IL, USA) was used to analyze the data of TPC, TFC, antioxidant capacities, and HPLC results. Analysis of variance (ANOVA) plus Duncan’s multiple comparisons was conducted to analyze the differences among different samples for TPC, TFC, antioxidant capacity, and the contents of phloridzin and trilobatin. Finally, Pearson correlation analysis was performed.

## 3. Results

### 3.1. Analysis of Single-Factor Tests

**Figure 1:**
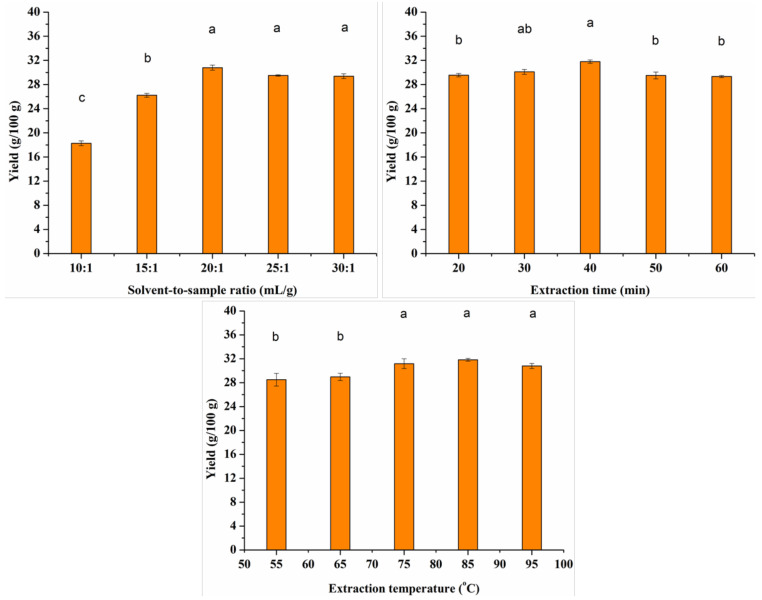
The result of single-factor tests for instant sweet tea powder yield (*n* = 3). Different letters (a, b, c) above the columns indicates significant differences (*p* < 0.05).

As shown in Figure 1, under the extraction condition of 85 °C and 30 min, the yield of instant sweet tea powder ranged from 18.28 to 30.80 g/100 g. The highest yield achieved the highest value at the solvent-to-sample ratio of 20:1. When the ratio was higher than 20:1, the yield decreased within a small range and largely remained stable.

Under the solvent-to-sample ratio of 20:1 and extraction temperature of 85 °C, the yield increased between 20 and 40 min and then decreased until 60 min. The yield of instant sweet tea extracted for 40 min was significantly higher than that extracted for other durations, reaching a value of 31.80 g/100 g. Considering the water efficiency and cost-effectiveness, the ratio of 20:1 mL/g was chosen as the best result.

Under the solvent-to-sample ratio of 20:1 and extraction time of 30 min, the yields with temperature from 75 to 95 °C were significantly higher than those in 55 and 65 °C. The highest yield value was 31.83 g/100 g, which was obtained at a temperature of 85 °C.

### 3.2. Response Surface Methodology (RSM) Experiments and Verification

**Figure 2:**
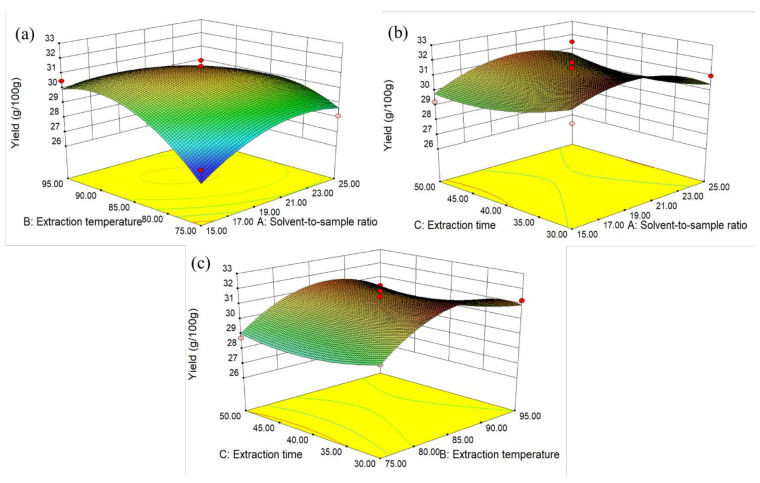
The three-dimensional response surface plots for the effects of solvent-to-sample ratio, extraction temperature, and extraction time on the instant sweet tea powder yield (*n* = 3).

As shown in Figure 2a, the yield of instant sweet tea powder increased with the increase in solvent-to-sample ratio (from 10:1 to 23:1 mL/g) and extraction temperature (from 75 to 90 °C) firstly, then slightly decreased when it approached the solvent-to-sample ratio of 25:1 mL/g and the temperature of 95 °C. According to Figure 2b, the yield increased with the solvent-to-sample ratio and reached its maximum at the ratio of 20:1, but the yield showed a downward trend with the extraction time from 30 to 40 min. Similarly, the yield also decreased with the extraction time from 30 to 40 min, while enhanced with the higher extraction temperature from the result of Figure 2c.

According to Table 1, the ANOVA analysis was also conducted, and the model was significant (*p* < 0.05), but the ‘Lack of Fit’ was not significant, indicating that the models were appropriate. In addition, the R^2^ was 0.8336, and the coefficient of variation (C.V.%) of instant sweet tea powder yield was 3.04, suggesting that only 16.6% of variance could not be explained by this model, indicating that the model was reliable. The contour plot of Figure 2a was elliptic, indicating that there was a significant effect of solvent-to-sample ratio and extraction temperature interaction (AB) on instant sweet tea yield, and this result was consistent with the surface quadratic model (Table 1).

The optimal extraction conditions by the result of RSM were the solvent-to-sample ratio of 19:1 mL/g, extraction temperature of 88 °C, and extraction time of 30 min. Under these conditions, verification experiments were conducted again, and the mean yields of instant sweet tea powder by predicted and actual experiments were 32.08 and 32.75 g/g, respectively, indicating that the model was reliable and accurate.

### 3.3. Analysis of TPC and TFC

**Figure 3:**
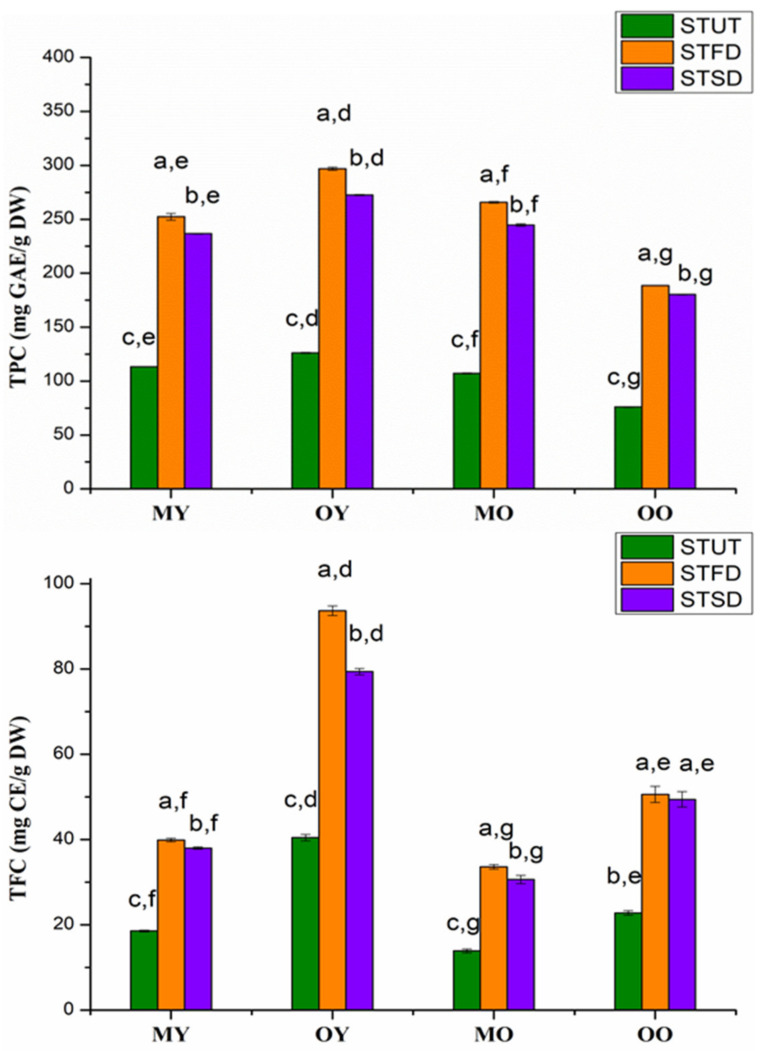
The total phenolic content (TPC) and total flavonoids content (TFC) of different samples (*n* = 3). Different letters (a, b, c) for the first label above the columns in each group indicates significant differences among STUT, STFD, and STSD (*p* < 0.05), respectively. Different letters (d, e, f, g) for the second label above the columns indicate significant differences among MY, OY, MO, and OO (*p* < 0.05), respectively.

As shown in Figure 3, the TPC of STUT, STFD, and STSD ranged from 76.04–126.2, 188.6−296.8, and 180.3–272.6 mg GAE/g DW, respectively. The TFC of STUT, STFD, and STSD ranged from 13.9–40.5, 33.6–93.1, and 30.7–79.4 mg CE/g DW, respectively.

Significant differences were found for the TPC and TFC among STUT, STFD, and STSD (*p* < 0.05), and the TPC and TFC of untreated sweet teas were significantly lower than those of instant sweet tea powders. The trends of TPC and TFC followed the orders: STFD > STSD > STUT.

Significant differences were also observed for TPC and TFC among different raw materials of sweet teas. The TPC and TFC of instant sweet teas produced by OY were significantly higher than those of other tea powders (*p* < 0.05). Overall, the TPC and TFC of instant sweet tea powders produced by young leaves were higher than those produced by old leaves.

### 3.4. Analysis of Antioxidant Capacity

**Figure 4:**
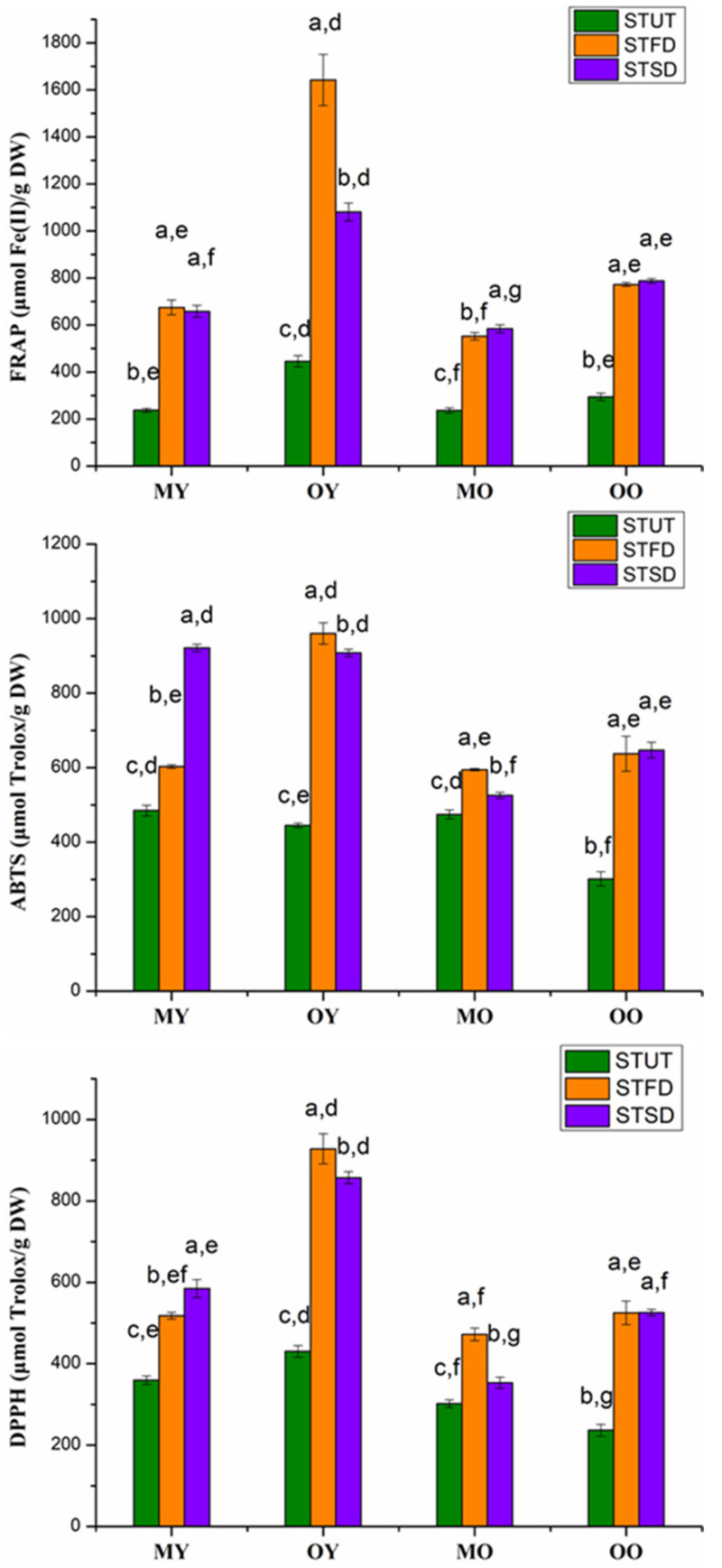
The antioxidant capacities of sweet teas and their instant teas (*n* = 3). Different letters (a, b, c) for the first label above the columns in each group indicate significant differences among STUT, STFD, and STSD (*p* < 0.05), respectively. Different letters (d, e, f, g) for the second label above the columns indicated significant differences among MY, OY, MO, and OO (*p* < 0.05), respectively.

As shown in Figure 4, the FRAP values of STUT, STFD, and STSD ranged from 237.1–446.0, 552.3–1642.6, and 584.2–1081.1 µmol Fe(II)/g DW, respectively. The DPPH values of STUT, STFD, and STSD ranged from 236.6–430.7, 472.4–928.0, and 353.4–584.7 μmol Trolox/g DW, respectively. The ABTS values of STUT, STFD, and STSD ranged from 301.00–485.0, 594.7–960.1, and 525.7–921.8 μmol Trolox/g DW, respectively. The FRAP, DPPH, and ABTS values had significant differences among STUT, STFD, and STSD (*p* < 0.05), and the FRAP, DPPH, and ABTS values of STUT were significantly lower than those of the other two instant sweet tea powders. However, no significant difference was observed for the FRAP, DPPH, and ABTS values between STFD and STSD produced from OO. The antioxidant capacity of instant sweet tea powders produced from OY was higher than those produced from other raw materials. Overall, the antioxidant capacities of instant sweet teas produced by young leave were higher than those produced by old leaves.

### 3.5. Identification of Phloridzin and Trilobatin Contents

As shown in Table 2, the phloridzin contents of STUT, STFD, and STSD were found to be significantly different, and the trend of phloridzin content followed the orders: STSD > STFD > STUT. The trilobatin contents of STUT, STFD, and STSD were also found to be significantly different, and the trend of trilobatin content followed the orders: STFD > STSD > STUT. The contents of phloridzin and trilobation in instant sweet teas (both freeze-dried and spray-dried) were approximately 2–3 fold greater than those in untreated sweet tea.

There were also significant differences in phloridzin and trilobatin contents among instant sweet teas produced by different raw materials. The contents of phloridzin were the lowest in MY, while the contents of trilobatin were the lowest in OO.

### 3.6. Correlation Analysis of Antioxidant Capacity, TPC, TFC, Phloridzin and Trilobatin Contents

In order to investigate the relationships among antioxidant capacity (FRAP, DPPH, and ABTS), TPC/TFC, and phloridzin and trilobatin contents, Pearson correlation analysis was employed (Table 3). Highly significant correlations were observed among antioxidant capacities (FRAP, DPPH, and ABTS), TPC, and TFC (*p* < 0.01), and a significant correlation was found between TPC and TFC (*p* < 0.05). However, no significant correlation was found between phloridzin/trilobation content and antioxidant capacities (*p* > 0.05).

## 4. Discussion

Although extraction condition studies have been reported by many researchers, the evaluation of the instant tea powders of *Lithocarpus litseifolius* [Hance] Chun is very limited. In our study, the yield of the instant sweet tea powder increased with the extraction temperature from 55 to 85 °C, and this result was similar to other studies [14,24], suggesting that a higher yield of instant tea powder might be associated with a higher phenolic content. As the temperature increases, the plant tissue softens and the viscosity and surface tension of water decrease, resulting in the dissolution of more phenolic compounds [25]. However, a slightly downward trend in the yield was observed at higher temperatures (e.g., 85 and 90 °C); this could be explained by some sensitive bioactive compounds, such as phenolic contents in sweet teas, decomposed under higher temperatures [26].

The maximum yield of instant sweet tea powder was obtained when extracting for 40 min and then declined in time. The result was consistent with a previous study that the total soluble solid of Roselle tea extracts reached a maximum after brewing for 40 min [27]. When the extracting time exceeded 40 min, the yield of instant sweet tea powders decreased, which may be associated with the degradation of bioactive compounds in sweet tea [28].

Figure 1 indicates that the change in solvent-to-sample ratio also influenced the total yield of instant sweet tea powder. In theory, the higher the solvent-to-sample ratio, the greater the yield of instant tea powder obtained [29]. However, the yield would not increase continuously but achieve maximum value and fluctuate around this value insignificantly [28]. In our study, the highest yield of instant sweet tea was found at the water-to-sweet-tea ratio of 20:1 mL/g, and no significant increase was observed when the ratio enhanced to 30:1 mL/g.

According to a previous study, the TPC and FRAP values of *Lithocarpus litseifolius* [Hance] Chun by maceration were 90.59 ± 0.67 mg GAE/g DW and 281.82 ± 9.21 µmol Fe(II)/g DW, respectively, which were within the TPC and FRAP ranges of STUT in our study. In addition, the mean values of phlorizin and trilobatin were 23.87 and 164.38 mg/g, respectively, and these results were very similar to the values of microwave-dried young leaves in our current study [30]. In another study, the yields of phloridzin and trilobatin were 34.76 ± 1.49 and 162.93 ± 1.73 mg/g, respectively [31], which were also close to our results. The FRAP, DPPH, and ABTS of MO and MY showed different trends among STFD and STSD; this could be explained by the fact that one or some compounds in extracts showed different responses for the FRAP, ABTS, and DPPH reagents, and this phenomenon has been similarly reported in previous studies [32,33].

We found that the TPC, TFC, and antioxidant activities of young sweet tea leaves dried in the oven (40 °C for 72 h) were higher than those of old leaves. This result was consistent with previous reports. The contents of TPC in aqueous young leaves after drying were higher than those in the old leaves of *Garcinia schomburgkiana* [34], and young guava leaves also showed higher antioxidant activities than old guava leaves [35]. In addition, the TPC and TFC in instant sweet teas by freeze-drying were higher than those in spray-dried instant sweet tea powders, and this could be attributed to the high temperature during spray-drying, leading to the degradation of phenolic substances.

There was a strong positive correlation between the TPC and the radical scavenging activity of instant tea, and the result was also found in other tea forms of *Lithocarpus polystachyus* [36], *Garcinia schomburgkiana* leaf extracts [34], and in white and green tea extracts [32]. The antioxidant capacities (FRAP, DPPH, and ABTS) were in significant correlation with each other, because these measures were essentially the same due to electron transfer capacities of compounds as antioxidants. In addition, the strong correlation between TPC and antioxidant capacities was associated with the total phenols assay; the Folin–Ciocalteu reagent was essentially an electron transfer based antioxidant assay [37].

The main dihydrochalcones of sweet tea and its instant tea powders were identified, and phlorizin and trilobatin were qualitatively and quantitatively analyzed. The spray-dried instant sweet tea showed higher phlorizin contents, while the freeze-dried instant sweet tea presented the highest content of trilobatin among different types of samples. A recently published study investigated the antioxidant capacities of trilobatin and phloridzin by DPPH-spiking test based on high-performance liquid chromatography (DPPH-spiking HPLC), and it was found that their antioxidant capacities were in the order of trilobatin > phloridzin [31]. Although a previous study reported that the antioxidant activities of sweet tea were closely related to dihydrochalcones (phloridzin, trilobatin, and phloretin), no significant correlation was found in our study, and this might be due to other components in sweet tea that contributed to higher antioxidant activities than these two compounds.

## 5. Conclusions

In order to obtain the maximum yield of instant tea powder by the young leaves of *Lithocarpus litseifolius* [Hance] Chun after freeze-drying, the optimal conditions for extraction were the solvent-to-sample ratio of 19:1 mL/g, extraction temperature of 88 °C, and extraction time of 30 min. The freeze-dried instant sweet tea produced by young leaves (prepared by oven) showed the highest antioxidant capacities compared with other raw materials and drying methods.

## Figures and Tables

**Table 1:** The analysis of variance (ANOVA) for response surface quadratic model.

Source	Sum of Squares	df	Mean Square	F Value	*p*-Value Prob > F	Significance
Model	29.19	9	3.24	3.90	0.0433	significant
Solvent-to-sample (A)	0.018	1	0.018	0.021	0.8886	
Extraction temperature (B)	3.78	1	3.78	4.54	0.0705	
Extraction time (C)	0.56	1	0.56	0.68	0.4374	
AB	4.79	1	4.79	5.75	0.0476	
AC	0.56	1	0.56	0.68	0.4382	
BC	0.004	1	4.79	0.005	0.9473	
A^2^	5.69	1	0.56	6.84	0.0347	
B^2^	12.08	1	0.004	14.51	0.0066	
C^2^	1.45	1	5.69	1.75	0.2280	
Residual	5.83	7	0.83			
Lack of Fit	4.31	3	1.44	3.78	0.115	not significant
Pure Error	1.52	4	0.38			
Cor Total	35.01	16				
*R* ^2^	0.8336					
*R* ^2^ *_Adj_*	0.8196					
C.V.%	3.04					

**Table 2:** The differences in phloridzin and trilobatin contents in samples.

	Sample	STUT (mg/g DW)	STFD (mg/g DW)	STSD (mg/g DW)
Phloridzin	MY	24.56 ± 0.14 ^c,f^	58.51 ± 0.67 ^b,g^	67.65 ± 1.98 ^a,g^
	OY	32.99 ± 1.56 ^c,e^	97.93 ± 1.53 ^b,e^	113.22 ± 3.37 ^a,f^
	MO	36.31 ± 1.32 ^c,e^	82.96 ± 1.77 ^b,f^	191.17 ± 3.53 ^a,d^
	OO	52.38 ± 1.73 ^c,d^	140.28 ± 5.28 ^b,d^	166.19 ± 5.70 ^a,e^
Trilobatin	MY	157.04 ± 2.90 ^c,e^	340.35 ± 6.83 ^a,d^	298.15 ± 1.63 ^b,e^
	OY	59.24 ± 0.13 ^c,f^	139.67 ± 1.84 ^a,e^	100.67 ± 0.50 ^b,f^
	MO	165.93 ± 0.41 ^c,d^	338.13 ± 4.60 ^a,d^	307.80 ± 2.78 ^b,d^
	OO	2.67 ± 0.17 ^b,g^	5.37 ± 0.32 ^a,f^	5.75 ± 0.31 ^a,g^

^a–c^ Different letters in the same row indicate significant differences among sweet tea and its instant sweet tea powders (*p* < 0.05). ^d–g^ Different letters in the same column indicate significant differences among different raw materials (*p* < 0.05).

**Table 3:** The correlation analysis of antioxidant capacities, TPC, TFC, phloridzin, and trilobation contents.

	FRAP	DPPH	ABTS	TPC	TFC	Phloridzin	Trilobatin
FRAP	1						
DPPH	0.959 **	1					
ABTS	0.838 **	0.915 **	1				
TPC	0.761 **	0.761 **	0.788 **	1			
TFC	0.982 **	0.953 **	0.789 **	0.681 *	1		
phloridzin	0.425	0.255	0.286	0.476	0.369	1	
trilobatin	−0.075	−0.006	0.188	0.531	−0.198	−0.058	1

* indicated statistical significance at *p* < 0.05, ** indicated statistical significance at *p* < 0.01.

## Data Availability

Data are available on reasonable request from the authors.
